# Supplementary material for: Are carbon emissions trading and green financial instruments synergistic? -Comprehensive quantitative research based on content analysis
Source: PLoS One. 2024 Mar 7;19(3):e0298601. doi: 10.1371/journal.pone.0298601 (PMC10919601; doi:10.1371/journal.pone.0298601)
Supplement: S1 Data — (ZIP) [file pone.0298601.s001.zip › Data/地级市Z_F工作报告.zip!/║■▒▒/╢⌡╓▌╩╨/└┤╫╘╣1⁄2╓┌║┼í╛┬φ┐╦╩2╛▌═°í┐_2010.docx]

各位代表：

　　现在，我代表市人民政府向大会作政府工作报告，请予审议，并请各位市政协委员和列席人员提出意见。

　　 一、2009年工作回顾

　　2009年是我市新世纪以来最困难的一年。面对国际金融危机的严峻挑战，全市上下深入贯彻落实科学发展观，按照省委、省政府和市委、市政府的决策部署，齐心协力，克难攻坚，经济企稳回升，社会和谐安定。全年实现地区生产总值313.6亿元，同比增长15.2%，增幅居城市圈第1位；财政总收入26.5亿元，增长4.9%；地方一般预算收入13亿元，增长21.6%，居第4位；全社会固定资产投资220.6亿元，增长47.1%；招商引资总额86.9亿元，增长35.8%；实际利用外资10514万美元，增长12.6%；全社会消费品零售总额126.7亿元，增长20.8%，居第3位；城镇居民人均可支配收入13408元，增长9.5%，居第2位；农民人均纯收入5718元，增长12.2%，居第3位；单位生产总值综合能耗、化学需氧量、二氧化硫排放量分别下降5.1%、7.5%、1.5%。除外贸出口受宏观形势影响未达目标外，本届人大二次会议确定的2009年主要预期目标均超额完成。

　　——强力推进项目建设，投资保持高位增长。出台了《关于扩大投资促进经济平稳较快增长的若干意见》，实施了重点项目派驻工作队和绿色通道制度，强化了对项目的协调督办，固定资产投资同比增幅为近年来最高。全力推进重点项目建设，实施亿元以上项目80个，同比增加11个，鄂钢宽厚板、鄂钢2200m3高炉、鄂州电厂二期、世纪新峰水泥二期、鸿泰钢铁三期、比克电池一期等项目竣工投产，星丰金属资源利用、华祥水泥、华工科技等项目开工建设。强力推进招商引资，举行各类招商活动20余次，签约项目152个，开工项目122个，华中自控、恒大六大中心、爱民制药等25个亿元以上项目先后开工，79个项目实现当年开工当年投产。积极抢抓中省投资，共争取四批新增中央投资项目274个，总投资21.5亿元，已下达新增中央投资2.83亿元，项目个数和投资额均创历史最好水平。

　　——有效服务企业运转，经济结构渐趋优化。编制了八大产业振兴和发展三年行动方案，制定了千亿元产业发展计划实施方案，实施了重点企业周监测、旬分析、月调度制度和“一企一策”扶持计划；大力推进中小企业成长工程，筹集1500万元增加中小企业担保公司担保基金；供电、石油石化等部门加强对煤电油气运的调度，保障了生产要素供应。企业结构进一步优化，实施工业技改项目69个，新增规模以上工业企业62家，无一家规模企业在金融危机中破产；销售收入过亿元的企业达到69家，同比增加 21家；新增内资企业139户、外商投资企业8户、私营企业809户。产品结构进一步优化，鄂钢宽厚板投产，鄂重产品首次打入美国市场；“枫树”被评为中国驰名商标，名牌产品实现销售收入、利润总额、税金占全市规模企业的比重均达45%以上。产业结构进一步优化，规模以上工业企业实现增加值176亿元，同比增长23.9%；现代服务业实现增加值68.22亿元，同比增长10.2%，成功举办了第十届梁子湖捕鱼旅游节；高新技术产业实现产值83.5亿元，同比增长25.1%。区域结构进一步优化，区级财政总收入、地方一般预算收入平均增幅分别高于全市18.6和14.9个百分点，葛店开发区成为武汉综合性国家高技术产业基地重要组成部分；鄂州开发区省级循环经济示范区建设加快推进，武汉港工业园全面开工建设。

　　——财贸金融平稳运行，支持发展力度加大。面对国际金融危机和国内财税政策调整的双重影响，深化财税改革，狠抓增收节支，有力扭转了骨干税源的大幅下滑，保障了收入平稳增长和重点支出需要。商贸流通业保持平稳较快发展，全面推进“万村千乡市场工程”，明堂等4家农贸市场进行标准化改造，蟠龙大市场开工建设；大力培植消费热点，积极开展“家电下乡”和“汽车、摩托车下乡”工作，全年发放财政补贴资金1950万元；粮食、供销、烟草等部门在开拓市场上取得新成效。银行、保险支持经济发展力度明显加大，积极搭建政、银、企融资平台，建立规模以上企业融资监测分析体系，全力为骨干企业、重点项目和中小企业提供信贷支持。存贷款均创历史最好记录，金融机构年末各项存款余额202.5亿元，比年初增加44.5亿元，同比增长28.2%；贷款余额99.6亿元，比年初增加21.2亿元，同比增长27%。

　　——积极开展先行先试，城乡一体化扎实推进。政策体系进一步完善，积极争取省委、省政府和省直部门大力支持，实施“厅市共建”，编制了《鄂州市城乡总体规划》，制定了城乡一体化总体方案和《城乡一体化三年行动计划》，出台了城乡一体化资金管理、中心村（新社区）规划建设、土地整理等三个管理办法和八个改革方案。平台建设进一步加快，市、区、镇三级投入8700万元加快特色镇建设；扎实推进中心村（新社区）示范点建设，试点村建设规划基本完成，基础设施逐步完善；启动了长港示范区建设，顺利实施土地整理、农业板块、公路水利、生态修复、环境综合整治等21个项目。产业基础进一步夯实，水产、蔬菜、畜牧、林果四大支柱产业加快发展，粮食等农产品产量保持较快增长，实现农业增加值43.98亿元，增长6.1%；农业产业化步伐加快，新增省级龙头企业3家、农业产业化组织6个、农民专业合作社69户。农村生产生活条件进一步改善，自来水主管网实现乡镇全覆盖，新增10.5万农村群众喝上自来水；完成7座小（一）型水库除险加固和洋澜湖泵站更新改造，硬化渠网135公里；新建通乡公路13.7公里、通村公路230公里；完成低丘岗地改造和高产农田建设9.1万亩；新增农机具6700台套；新建沼气池8187口；推进第二轮整村扶贫开发，减少贫困人口8150人。

　　——深入推进改革创新，“两型社会”建设步伐加快。进一步完善规划体系，《武汉城市圈“两型社会”建设鄂州示范区改革发展规划纲要》通过国家发改委组织的专家论证，《鄂州长江经济带发展规划》、《大梁子湖运动休闲生态旅游度假区规划》等重大规划抓紧编制。强力推进武汉新港鄂州港区建设，三江港被列为武汉新港核心港区，《武汉新港三江港区控制性详细规划》修编全面展开，三和管桩综合码头、鄂钢工业港、超凡物流园综合码头、星丰项目配套码头等项目启动。强化生态环境保护，抓紧编制《梁子湖生态环境保护规划（2009-2013）》，《鄂州市洋澜湖水环境治理工程可行性研究报告》获省发改委批复，我市作为代表参加第十三届世界湖泊大会，交流梁子湖保护经验；实施了葛店开发区和梁子岛污水处理、鄂州电厂脱硫、鄂钢烧结脱硫等一批节能减排重点工程，对辖区内长江沿岸各类排污口进行了拉网式检查，全面完成省政府下达的9个专项整治任务，淘汰小水泥、小炼铁等落后产能136.6万吨，空气优良天数占全年的96.7%。

　　——加快基础设施建设，交通城建取得突破。交通建设超常规发展，完成投资近7亿元，同比翻4番，交通环境大为改善。316国道鄂州段改造、省道樊湖线改造、葛湖线路基建设等工程相继完成，樊口大桥建成通车，汉鄂高速、城际铁路、火车站改造、武九线改造等项目顺利推进。城市建设全面提速，十大市政工程全部启动，完成投资4亿元。“环湖”城市亮点工程和“一线串珠”的沿江文化走廊相继启动，洋澜湖综合治理全面实施，完成主城区12公里道路刷黑和30条背街小巷改造。投融资模式实现新突破，市城投公司筹融资总额达8.41亿元，采取BT、BOT方式建设城市中轴线、樊口污水处理厂等项目。城市管理水平不断提高，城市生活垃圾填埋场投入使用，拆除违法建设18.8万平方米，新建公厕42座，建成区绿地率、绿化覆盖率、人均公共绿地面积分别达到36.9%、42.3%、11.3平方米，市容环境得到改善。“四城联创”取得突破，国家卫生城、环保城、文明城创建进展顺利，国家园林城创建顺利通过专家组现场考核检查和住建部综合评审。

　　——努力保障和改善民生，社会保持和谐稳定。千方百计加大投入，“十件实事”全面完成，筹集到位资金9.6亿元，占年筹资计划的128%。扎实做好创业促就业工作，共发放小额担保贷款8004万元，城镇新增就业2.1万人，劳动力转移培训1.33万人，我市被确定为全省首批创建创业型城市。全力推进社会保障工作，五项社会保险新增扩面4.95万人次，全年享受社会保险待遇121万人次，支付社会保险金额6.29亿元；大力推进城乡医保一体化，综合参保率90.5%，居全省领先水平；发放城乡低保金7035万元，发放医疗救助金527.9万元，全国低保和临时救助工作会议在我市召开。筹集5500万元新建的城市福利中心投入使用。全力推进社会事业发展，教育事业加快发展，启动了农村中小学布局调整工作，筹资2800万元建成22所农村寄宿制初中，素质教育整体推进，教育质量全面提高，义务教育学校教师绩效工资基本到位，武汉城市圈（鄂州）职教园区获省政府批准建设，鄂州大学建成国家级实训基地1个、省级实训基地2个。科技事业快速发展，取得省级科技成果10项，专利申请量突破500件，我市被评为全国科技进步先进城市。加强精神文明建设，社会科学、文学艺术、新闻出版、广播电视等事业进一步繁荣。三国吴都博物馆主体工程封顶，新建了市少儿图书馆，完成6个乡镇综合文化站维修改造工程，率先在全省启动农村有线电视“三网融合”、实现“农家书屋”村级全覆盖，群众性文体活动蓬勃开展，大型舞蹈诗《吴都风华》成功上演。医药卫生体制改革全面启动，完成了62家村卫生室、7所乡镇卫生院和2个社区卫生服务中心建设，市中心医院、市中医医院和市妇幼保健院改扩建工程相继启动或完成，科学有序开展甲型H1N1流感防控和救治工作。人口计生工作质量明显提高，人口自然增长率控制在6‰以内。住房保障工程加快实施，建设廉租房1643套，完成经济适用房100套，发放住房租赁补贴315万元。发放住房公积金个人贷款2.6亿元，使1662户低收入家庭圆了住房梦。投入3082万元，援建了四川省汉源县第一中学扩建等五大工程，圆满完成了对口援建任务。普法教育深入推进，社会治安综合治理得到加强，连续三年实现“命案全破”，群众安全感不断提升。市场监管力度加大，工商、质监和食品、药品市场监管进一步加强。深入开展重点行业和重点领域的安全生产隐患排查和治理，积极做好消防安全工作，大力开展治超治限工作，努力提升应急管理水平，安全生产保持平稳态势。信访工作得到加强，各级领导实行包案下访，有力地促进了社会稳定。

　　——切实转变政府职能，发展环境不断优化。深入开展学习实践科学发展观活动，进一步提高了科学发展的自觉性和主动性。民主法治建设不断加强，2009年共办理人大建议和政协提案432件，办复率100%，满意和基本满意率95%以上。全面落实《鄂州市人民政府工作规则及相关重要规程》，邀请市人大代表、市政协委员、市民代表参加市政府常务会议，科学化、民主化决策机制不断完善。积极稳妥推进政府机构改革，优化行政职权运行流程，全面清理行政审批事项，审减市属许可项目135项、非行政许可事项25项。严格规范招投标、土地出让、政府采购、国有资产管理，强化政府投资项目审计，大力实施行政问责和效能监察。稳妥推进国企改革收尾工作。严格实行重大行政处罚备案审查制度，进一步完善收费明白卡和企业宁静日制度，深入开展文明执法教育、行风评议和发展环境万人评议活动，加大了对损害经济发展环境行为的查处力度，市直各部门服务发展的意识进一步增强，行政执法水平稳步提升。加快推进政务公开和电子政务建设，进一步延伸行政服务领域与层次，初步形成自上而下的四级行政服务体系，人民群众满意度不断提高。

　　与此同时，统计、物价、民族宗教、外事侨务、对台、保密、档案方志、老龄、妇女儿童、残联、邮政、通讯、无线电管理、气象、人防和国防动员、民兵建设等工作都取得了新的成绩。

　　各位代表！过去的一年，挑战非同寻常，成绩来之不易。这是全市人民团结奋斗的结果；是各方面鼎力支持的结果；也得益于历届市委、市政府打下的良好基础。借此机会，我谨代表市人民政府向辛勤工作在各个岗位上的广大干部群众，向各位人大代表、政协委员，向各民主党派、工商联、无党派人士、人民团体和社会各界人士，向为鄂州发展做出贡献的各位老同志，向驻军指战员、武警官兵以及所有来鄂州的投资者、劳动者和海内外朋友表示崇高的敬意和衷心的感谢！

　　在充分肯定成绩的同时，我们也清醒地认识到面临的困难和问题。一是经济回升的基础还不稳固。民间投资意愿不强，消费需求增速不快，外贸出口形势严峻；二是结构性矛盾仍很突出。经济发展对少数行业、企业依赖性仍然过大，经济整体抗风险能力还不强，转变经济发展方式任务艰巨；三是财政增收节支压力较大。一方面，受金融危机和结构性减税政策双重影响，财政收入增幅明显放缓。另一方面，为拉动经济增长和保障民生，支出增长较快，给财政资金调度带来较大压力；四是经济发展环境仍需改进。部分单位的工作效能和依法行政水平仍待提高，等等。对这些问题，我们将以高度的责任感和使命感，采取更加有力的措施，认真加以解决。

　　二、2010年工作安排

　　2010年是“十一五”规划的收官年，是实现“两个率先”的关键年，做好今年的政府工作，具有十分重要的意义。今年政府工作的指导思想是：认真贯彻落实党的十七大、十七届三中、四中全会、中央和全省经济工作会议精神，深入贯彻落实科学发展观，以转变发展方式为主线，以“两型社会”示范区和城乡一体化试点为抓手，以优化结构为重点，以改革开放为动力，以改善民生为出发点和落脚点，变后危机时期为发展机遇期，咬紧发展不放松，努力实现经济较快增长和社会和谐稳定。主要预期目标是：生产总值增长13.5%，全社会固定资产投资增长30%，地方一般预算收入增长15%，规模以上工业增加值增长23.5%，招商引资总额增长35%，实际利用外资增长8%，外贸出口增长10%，全社会消费品零售总额增长17%，城镇居民人均可支配收入增长10%，农民人均纯收入增长10%，城镇新增就业2万人，城镇登记失业率控制在4.5%以内，人口自然增长率、居民消费价格涨幅、万元生产总值综合能耗、二氧化硫和化学需氧量排放量、亿元生产总值生产安全事故死亡人数均控制在省下达目标以内。

　　围绕以上目标任务，我们将着重抓好以下重点工作。

　　（一）一手抓项目建设，一手抓招商引资，着力推进投资拉动增长。一是着力抓好百个重点项目建设。对续建和新开工的119个亿元项目定目标、定时限、定责任，千方百计加快进度，确保年度投资210亿元。重点加快程潮铁矿新选矿厂、爱民制药、华祥水泥、武汉港工业园等续建项目的建设进度，力争早日竣工投产；推进鄂钢“十一五”技改二期、武钢平煤焦油、星丰金属资源利用、湖北进口资源再利用、武汉新港三江港铁路物流中心、华工科技园等一批重大项目，确保按期开工建设。二是着力抓好百个政府投资项目申报争取工作。根据中央和省经济工作会议确定的投向，明确主攻目标和方向，做好已策划的141个中央投资项目和767个“两型社会”建设项目的前期策划申报工作，争取落实一百个中央投资项目。及早谋划“十二五”发展规划，加强与国家、省规划对接，围绕国家重点扶持的新能源、信息网络、新材料、生物医药等产业和行业，筛选和储备一批重大项目，争取更多项目纳入中央和省规划笼子。三是着力抓好百个招商引资重点项目。动态确定百个招商引资洽谈和签约项目，定期协调督办，扎实跟进。认真落实优惠政策，严格兑现招商奖励，改进招商引资方式，前移招商阵地，巧搭招商平台，广辟信息渠道，精心策划招商活动，全面推进与珠三角、长三角、环渤海等发达地区的合作与联系，紧盯中字号、国字号和世界500强，着力引进一批投资规模大、科技含量高、辐射能力强、成长前景好的项目。四是着力优化项目建设环境。切实抓好项目服务保障，开展项目建设环境专项整治活动。市财政继续安排500万元资金做好重大项目库建设和项目前期工作。强化领导联系责任制和工作队帮扶制，切实帮助项目建设单位解决征地、拆迁、资金等难题。努力实现竣工项目保投产、投产项目保运行、运行项目保效益。

　　（二）一手抓产业壮大，一手抓结构调整，着力推进经济提档升级。一是进一步壮大市场主体。实施大企业培育计划。充分利用国家和省产业振兴计划，重点支持鄂钢、球团厂、电厂、程潮铁矿等企业做优做强，扶持鸿泰钢铁、吴城钢铁、波尔容器、世纪新峰、新鄂重等一批中型企业提档升级。继续大力实施中小企业成长工程。建立和完善信用担保、技术创新等社会化服务体系，完善中小企业成长工程的考核奖励办法，落实关于促进中小企业发展的系列措施，财政列支100万元设立金融机构扩大中小企业贷款奖励基金，推动成长型中小企业发展壮大。二是进一步抓好结构调整。推进工业结构转型升级。大力改造提升冶金、建材、服装等传统支柱产业。切实加大工业企业技改力度，力争中小企业技改项目新增增加值10亿元。大力发展生物医药、电子信息、船舶、太阳能等新兴产业，组织实施各类科技专项，争取实现高新技术产业产值100亿元，促进我市产业素质整体提升。三是进一步抓好园区建设。重点加强葛店开发区、鄂州开发区、花湖开发区、鄂城新区、华容创业园、三江港经济区等六个园区建设。新建项目原则上一律进园区，各园区间实行错位发展，避免恶性竞争、资源浪费。进一步完善园区功能。加快葛湖路等道路建设，不断提升平台承载力。加速推动梧桐湖新区的水、电、路等基础设施建设，将梧桐湖新区打造成全市新的发展平台。四是进一步推进服务业跨越发展。落实服务业税收扶持政策，加强对服务业的用地支持，重点发展现代物流业、旅游业、金融保险业、信息服务业。加快建设三江港区、主城区和花湖工贸新城三大物流基地，加快鄂州港区和武汉港区合作开发步伐。加大“一都两湖”基础设施建设力度，抓好庙岭镇、梁子岛旅游名镇建设。办好第十一届梁子湖捕鱼旅游节，打造大梁子湖运动休闲旅游度假区。加速实施凤凰山庄五星级改造、大鹰山五星级产权式酒店项目。

　　（三）一手抓促进消费，一手抓扩大出口，着力推进经济协调发展。一是大力推进居民消费持续繁荣。采取多种措施，培育消费热点，扩大消费需求。抓好特色商业街、专业市场和高端商场、酒店建设，提升城市对消费的引导能力。加强农村市场体系建设，扩大“万村千乡市场工程”覆盖范围，继续做好“家电下乡”和“汽车以旧换新”工作，积极培育农村消费市场。加快激活房地产市场，规范房地产市场秩序，积极促进住房消费。继续推进非公经济单位住房公积金制度，改善职工住房条件。加强市场运行调节，改善消费环境，让群众放心消费。二是大力推进出口增长。进一步调整优化出口商品结构，增强出口产品国际竞争力。全面落实出口鼓励政策，建立重点出口企业和项目联系制度，组织出口企业参加国内外各类展销活动。努力开拓南美、东南亚、非洲等新兴国际市场，巩固纺织服装产品出口，扩大机电、生物医药和农副产品出口。建立健全出口企业融资贷款和质押担保渠道，鼓励和支持企业发展自营进出口业务。三是大力推进对外合作交流。鼓励和支持企业开展跨国投资和经营，开展对外工程承包和劳务合作，拓展发展空间。办好对外劳务服务中心，扩大劳务输出。加大对外人才交流力度，大力引进人才。通过科技考察、学术交流、技术培训等形式，借鉴国外先进经验，提高企业自主创新能力。进一步拓宽与国外友好城市的交流渠道，开展多领域合作。

　　（四）一手抓村镇建设，一手抓产业培育，着力推进城乡一体化。一是加快推进城镇建设工程。把国土整理与其它工程项目有机结合，加快小城镇建设管理的功能配套和提档升级。市、区、镇三级继续筹资6000万元加快十个特色镇建设，重点支持特色镇完善功能配套和拉开骨架，建设各类专业市场、产业园区或农民创业园。大力实施村庄环境整治工程，改善农村人居环境。二是加快推进百里长港统筹城乡示范区建设。实施农村新社区建设工程，逐步引导农民向农村新社区集中。完成示范区整体发展规划、各专项规划和村镇分规划。推进示范区基础设施建设，启动百里长港生态修复工程，开展城市公交“进镇入村”试点。跟踪落实“厅市合作”协议，争取省直部门的再支持。三是加快推进农业产业化工程。深入贯彻中央1号文件精神，以提高农民收入为主线，大力发展现代农业，切实抓好农业板块建设，着力推进水产、蔬菜、畜牧、林果四大支柱产业加快发展。大力实施农业产业化龙头工程，加快培育和发展农业专业合作组织，建设农产品加工园区，提高农业产业化水平。切实抓好农产品质量安全工作。大规模开展中低产田改造和低丘岗地整理，加快实施农田水利基础设施建设工程。实施“十镇百村”市场建设工程，大力发展劳务经济。坚持开发式扶贫方针，稳步推进整村扶贫开发工作。积极发展休闲观光农业，进一步拓宽农民增收渠道。

　　（五）一手抓体制改革，一手抓机制创新，着力推进“两型社会”建设。一是切实抓好政府机构改革。组织实施好市区两级政府机构改革，认真制定政府部门三定方案，抓好改革过程中的检查督办，确保改革稳步推进。二是深入推进财税金融体制改革。积极创新财政体制机制，加快公共财政体系建设步伐。推进综合预算、国库集中支付、政府采购改革，不断提高财政管理水平和效能。推进农村综合改革，完善农村义务教育改革和村级组织运转经费保障机制，推进农村公益事业“一事一议”财政综合奖补试点工作。继续贯彻适度宽松货币政策，积极筹建市金融办，引导、鼓励、支持各类金融机构改革创新、拓展业务，努力保持金融平稳运行。充分发挥市中小企业担保基金作用，进一步加大对小额贷款和中小企业担保贷款的支持力度。鼓励有条件的企业参与直接融资，积极支持大通公司、武大有机硅和湖北顾地塑胶等企业上市。进一步加强信用环境建设，促进银企合作，努力实现经济和金融的良性互动。三是稳妥推进其它各项改革。以推进公立医院改革试点和建立国家基本药物制度为重点，稳妥推进医药卫生体制改革。积极推进事业单位岗位设置和绩效工资改革。稳妥推进户籍制度、土地管理和使用制度等重点领域和关键环节的改革。切实做好国企改革收尾工作。四是加快建设“两型社会”示范区。争取省委、省政府早日批复《武汉城市圈“两型社会”示范区改革发展规划纲要》。推动葛店开发区与武汉东湖高新区全方位融合，大力打造国家级高技术产业基地。加快建设青（山）阳（逻）鄂（州）大循环经济区鄂州开发区循环经济示范区等重点项目，大力发展低碳经济和循环经济。推进省级梁子湖“两型社会”示范区建设，进一步抓好生态环境保护。切实加强对重点排污单位的监管，确保环保设施发挥减排效益。认真落实环境准入制度，着力强化环保执法，全面完成“十一五”主要污染物总量减排目标。

　　（六）一手抓主城升级，一手抓新城拓展，着力推进城市功能提升。今年市政府将多方筹资实施基础设施建设“十大工程”，不断完善城市功能。一是切实抓好城市建设。主城区重点推进一湖（洋澜湖生态公园）、二线（迎宾大道南北中轴线、东西中轴线）、三区（中心城区、城东鄂城新区、城西鄂州开发区）、四山（西山、雷山、葛山、莲花山）建设。继续实施好配套的路桥工程、园林绿化工程、道路刷黑工程和背街小巷改造工程。推进葛华科技新城、红莲湖旅游新城和花湖工贸新城的市政配套设施建设。二是大力提升鄂州交通枢纽地位。加快推进交通投资公司的运作，做好交通投融资平台。加快推进汉鄂快速通道等重点公路建设和改造，推进两条城际铁路和鄂州火车站等铁路工程建设。加快鄂州水运口岸开放进程，做好《武汉新港鄂州港区详细规划》评审及报批工作，确保鄂钢工业港码头、超凡物流园综合码头、星丰金属资源码头建设取得实质性进展。三是强力推进“四城联创”。创新城市管理机制，探索推行城市管理行政执法改革。做好国家园林城创建的后续工作，切实推进国家卫生城、环保城、文明城创建，确保创建活动取得成效。

　　（七）一手抓社会事业，一手抓民生改善，着力推进和谐发展。一是大力发展社会事业。积极引进各类人才，切实做到“人才优先”。整合城乡义务教育资源，调整农村中小学布局，实施中小学校舍安全工程，推进教育均衡发展，加快职业教育发展，全面提高教育质量。积极促进文化产业发展，继续抓好乡镇综合文化站、“农家书屋”建设，进一步加强文化体育、新闻出版市场监管，广泛开展群众性文化体育活动，丰富城乡居民精神文化生活。推进村村开通无线广播、有线电视工作。加强公共卫生医疗服务体系建设，提高医疗保障水平，逐步解决群众看病难、看病贵问题。做好第六次人口普查工作。综合治理出生人口性别比偏高问题，稳定低生育水平。二是切实解决民生问题。全面落实税费减免、小额担保贷款、资金补贴等政策，继续实施劳动密集型企业贷款贴息，鼓励全民创业，争创全省创业型城市。做好农村劳动力转移培训和困难群众、零就业家庭就业工作。整合城乡低保资源，推进城乡社会救助体系建设，解决好失地农民生活保障和就业难问题。做好法律援助工作。办好梁子湖区新型农村社会养老保险试点。三是全力维护社会安全稳定。全力抓好化解社会矛盾、创新社会管理和公正廉洁执法三项重点工作。拓宽社情民意的诉求渠道，加强基层维稳工作，促进社会和谐稳定。提高自然灾害应急处置能力。高度重视安全生产，严格落实安全生产责任制，建立重大危险源（点）监控网络，加大重点行业、重点领域安全隐患的排查整改力度，严格查处安全生产事故，提高应急救援能力。加大对工业产品和食品药品安全监管力度，确保人民群众生命财产安全。四是继续做好十件实事。多方筹资办好扩大就业再就业、改善困难群众住房条件、拓展公共卫生服务、改善办学条件、推进城乡公共服务均等化、改善农民生活条件、改善农村生产条件、便利市民生活、改善城镇居民生活环境、建设四级行政服务体系十件实事。

　　（八）一手抓依法行政，一手抓提升效能，着力推进政府自身建设。一是努力提高科学决策能力。健全重大事项决策的规则和程序，市政府重大行政决策依法向市人大及其常委会报告，向市政协通报并协商。重大行政决策的立项、论证和评估邀请市人大代表和市政协委员参与。市政府在作出重大决策前，根据需要，通过多种形式直接听取民主党派、决策咨询部门、专家学者、基层群众等方面的意见和建议。健全专家咨询制度和社情民意反馈制度。积极发展电子政务，建立科学的政府信息公开机制。二是努力提高依法行政的执行能力。深入贯彻国务院《全面推进依法行政实施纲要》和《关于加强市县依法行政的决定》，继续完善依法行政体系，严格执行规范性文件的清理和后评价制度，坚持一案一评，加强行政层级监督和行政问责，提高依法行政质量。自觉接受人大的法律监督和政协的民主监督，认真办理人大代表议案、建议和政协委员提案，发挥民主党派、工商联、无党派人士的参政议政作用，支持工会、共青团、妇联等群团组织参与社会管理和公共服务。三是努力提高公共服务能力。坚持以人为本，进一步提高政府的公共服务能力，加快建立完善四级行政服务体系。按照“便民、高效、廉洁、规范”的总要求，积极推行“一楼式办公、一窗式收费、一站式服务”运行模式，分层级设立市行政服务中心、区（开发区）行政服务分中心、街道（乡镇）便民服务中心（大厅）、社区（村）便民服务中心（室）。到今年六月底前，基本完成市、区（开发区）、街道（乡镇）、社区（村）四级行政服务体系建设，形成较为完善的行政服务体系。四是切实加强廉政建设。全面落实党风廉政建设责任制，强化政府各级公务员队伍廉洁自律教育，全面实施腐败风险预警防控，严肃查处各类腐败案件。积极推进源头治腐工作，进一步规范权力运行，深化相关改革，完善约束机制，不断加强行政监察和审计监督。坚持艰苦奋斗、勤俭节约，努力降低行政成本，建设节约型机关，以良好形象赢得人民群众的信任和支持。

　　各位代表，今年是“十一五”规划的最后一年，也是“十二五”规划的衔接之年，鄂州改革发展稳定的任务十分艰巨。让我们紧密团结在以胡锦涛同志为总书记的党中央周围，在省委、省政府和市委的领导下，同心同德，开拓进取，再鼓干劲，再创新绩，努力开创鄂州更加美好的明天！
